# Individual lipid transfer proteins from *Tanacetum parthenium* show different specificity for extracellular accumulation of sesquiterpenes

**DOI:** 10.1007/s11103-022-01316-2

**Published:** 2022-10-18

**Authors:** Arman Beyraghdar Kashkooli, Aalt D. J. van Dijk, Harro Bouwmeester, Alexander van der Krol

**Affiliations:** 1 Laboratory of Plant Physiology, Wageningen University and Research, Droevendaalsesteeg 1, 6708 PB Wageningen, The Netherlands; 2 Applied Bioinformatics, Bioscience, Plant Sciences Group, Wageningen University & Research, Wageningen, The Netherlands; 3 Present Address: Plant Hormone Biology Group, Swammerdam Institute for Life Sciences, University of Amsterdam, Science Park 904, 1098 XH Amsterdam, The Netherlands; 4 Department of Horticultural Science, Faculty of Agriculture, Tarbiat Modares University, PO Box 14115-336, Tehran, Iran

**Keywords:** Heterologous expression, Lipid transfer protein, Sesquiterpenoids, Extracellular transport, Export assay, Exclusion assay, Specialized metabolism, Transport

## Abstract

**Key message:**

A highly specialized function for individual LTPs for different products from the same terpenoid biosynthesis pathway is described and the function of an LTP GPI anchor is studied.

**Abstract:**

Sequiterpenes produced in glandular trichomes of the medicinal plant *Tanacetum parthenium* (feverfew) accumulate in the subcuticular extracellular space. Transport of these compounds over the plasma membrane is presumably by specialized membrane transporters, but it is still not clear how these hydrophobic compounds are subsequently transported over the hydrophilic cell wall. Here we identified eight so-called non-specific Lipid transfer proteins (nsLTPs) genes that are expressed in feverfew trichomes. A putative function of these eight nsLTPs in transport of the lipophilic sesquiterpene lactones produced in feverfew trichomes, was tested in an in-planta transport assay using transient expression in Nicotiana benthamiana. Of eight feverfew nsLTP candidate genes analyzed, two (TpLTP1 and TpLTP2) can specifically improve extracellular accumulation of the sesquiterpene costunolide, while one nsLTP (TpLTP3) shows high specificity towards export of parthenolide. The specificity of the nsLTPs was also tested in an assay that test for the exclusion capacity of the nsLTP for influx of extracellular substrates. In such assay, TpLTP3 was identified as most effective in blocking influx of both costunolide and parthenolide, when these substrates are infiltrated into the apoplast. The TpLTP3 is special in having a GPI-anchor domain, which is essential for the export activity of TpLTP3. However, addition of the TpLTP3 GPI-anchor domain to TpLTP1 resulted in loss of TpLTP1 export activity. These novel export and exclusion assays thus provide new means to test functionality of plant nsLTPs.

**Supplementary Information:**

The online version contains supplementary material available at 10.1007/s11103-022-01316-2.

## Introduction

**Fig. 1:**
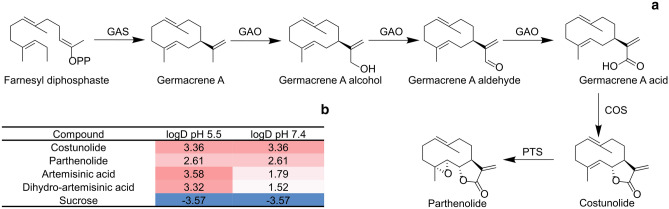
Biosynthetic pathway of parthenolide and partitioning coefficient of some sesquiterpene lactones and sucrose. **a** Biosynthesis pathway of parthenolide. *GAS*: germacrene A synthase; *GAO*: germacrene A oxidase; *COS*: costunolide synthase and *PTS*: parthenolide synthase. **b** log D (partitioning of a chemical compound between an organic versus aqueous phases; corrected for the pH) of costunolide, parthenolide, (dihydro)artemisinic acid and sucrose. The lower and/or more negative the number, the higher the partitioning in the aqueous phase (= better water solubility)

In many plants specialized metabolites are synthesised in specialised cells and subsequently transported over the plasma membrane (PM) and cell wall for accumulation in the extracellular space. The bioactivity of many of these metabolites, such as for example sesquiterpenoids, is based on their chemical reactivity with proteins and other cellular components, and sequestration into the extracellular space is likely required to avoid that these compounds react with the plant’s own cellular components. The medicinal sesquiterpenoids and their precursors produced by (for instance) feverfew and *Artemisia annua* are lipophilic as indicated by the chemical distribution coefficient [LogD (Leo et al. 1971)] (Fig. 1b). Such lipophilic compounds may therefore show low spontaneous diffusion over the hydrophilic cell wall once transported over the PM of the glandular trichome cells, especially since the pH of the cell wall is lower than that of the cytosol. Here we studied the extracellular sequestration of two medicinal sesquiterpenoids produced by feverfew: costunolide and parthenolide, both of which are active against ovary, prostate, breast and oral cancer as well as leukaemia (Choi et al. 2005; Zunino et al. 2007; Hsu et al. 2011; Yang et al. 2011; Yu et al. 2015). In feverfew these compounds are produced in the glandular trichomes on the ray florets and ovaries of the flowers and are secreted and accumulated in the subcuticular space of these trichomes (Majdi et al. 2011).

Biosynthesis of costunolide and parthenolide from the precursor germacrene A (GA) takes place in the cytosol and on the ER membrane by a sesquiterpene synthase and cytochrome P450s (Fig. 1a). The mechanism responsible for the sequestration of costunolide and parthenolide into the extracellular space of the trichomes is not known. Transport of these compounds within the cell could be by spontaneous or vesicular transport (Moreau & Cassagne 1994; Ting et al. 2015). Subsequent transport over the PM may occur through the action of different membrane transporters. For instance, two *A. annua* ABCg-type PM transporters have been identified that are involved in the transport of (dihydro)artemisinic acid [(DH)AA] over the PM. We postulate that ABCg-type transporters are also involved in the secretion of costunolide and parthenolide in feverfew. However, once on the outside of the PM the hydrophobic sesquiterpenoids are confronted with the hydrated polysaccharides of the cell wall which forms a hydrophilic environment suggesting that transport of sesquiterpenoids over the cell wall must somehow be facilitated. Indeed, also modelling suggests that the actual observed dynamics of (hydrophobic) volatiles emitted by plants requires the involvement of something that facilitates passage over the hydrophilic cell wall (Widhalm et al. 2015). For the product of the artemisinin biosynthesis pathway (DH)AA it was shown that an *A. annua* NsLTP (AaLTP3) enhances sequestration of (DH)AA into the apoplast (Wang et al. 2016). Lipid transfer proteins (LTPs) are small proteins usually with a size of 7–9 KDa, which contain a highly conserved 8 cysteine motif (Kader 1996). These cysteines connect to each other in four disulphide bridges resulting in four α-helices which together form a hydrophobic cavity (Shin et al. 1995) which in in vitro assays can bind a range of lipid molecules (de Oliveira Carvalho & Gomes 2007). NsLTPs contain a secretion signal and are predicted to be secreted into the cell wall of plants. Indeed, extracellular localisation has been confirmed for multiple LTPs in several different plant species (Sterk et al. 1991; Coutos-Thevenot et al*.* 1993; Choi et al. 2005; Wang et al. 2016).

The genes for parthenolide biosynthesis (*TpGAS*, *TpGAO*, *TpCOS* and *TpPTS*) in feverfew have been identified (Fig. 1a) and were characterized by expression in yeast and *Nicotiana benthamiana* (Liu et al. 2011, 2014; Majdi et al. 2011). Reconstitution of the parthenolide biosynthesis pathway in *N. benthamiana*—using transient expression—results in the accumulation of conjugates of costunolide and parthenolide with glutathione (GSH) or cysteine (Cys), while only very low levels of free costunolide and parthenolide are detected (Liu et al. 2011, 2014; Majdi et al. 2011). Here we demonstrate that the presence of free costunolide and parthenolide in such transient expression assays is due to their accumulation in the apoplast, thus escaping intracellular conjugation. In the background of this low intrinsic export activity for costunolide and parthenolide in *N. benthamiana* leaves, we tested eight feverfew Lipid transfer proteins (TpLTP*s*) for their effect on the accumulation of free costunolide and parthenolide. The *TpLTP* genes were selected based on their co-expression pattern with costunolide/parthenolide biosynthetic pathway genes over six developmental stages of feverfew flowers (Majdi et al. 2011). The activity of each TpLTP was tested in combination with the costunolide and parthenolide biosynthesis pathway by transient co-expression of the *TpLTPs* and costunolide/parthenolide biosynthesis genes in *N. benthamiana*. Our results suggest that in feverfew glandular trichomes, so-called Non-specific Lipid transfer proteins (NsLTPs) have acquired a specialized function by aiding sesquiterpene lactone transport over the cell wall. Implications for metabolic engineering of (sesqui)terpenes and potential wider implications for the role of NsLTPs in plants are discussed.

## Materials and methods

### RNA-sequencing and expression data analysis

**Fig. 2:**
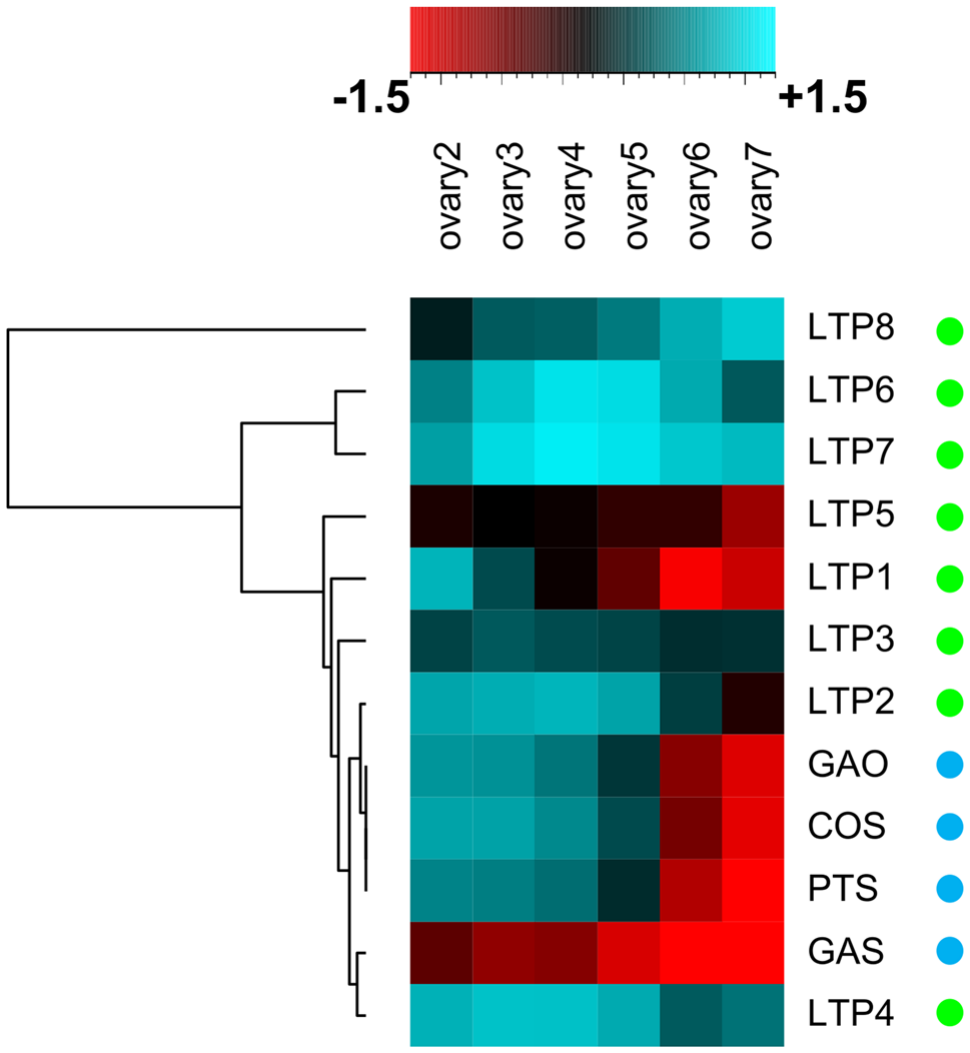
Expression pattern of feverfew parthenolide biosynthesis pathway genes (blue circles) and Lipid Transfer Proteins (LTPs) (green circles). Complete linkage cluster analysis of feverfew sesquiterpene lactone biosynthesis pathway genes and LTPs. Heatmap representation of normalized log_2_ transformed expression data (− 1.5 to + 1.5) of genes during six developmental stages of feverfew flowers. Each data point is the mean value of the three measured biological replicates. Green circles represent LTPs whereas blue circles represent feverfew sesquiterpene lactones biosynthesis pathway genes

RNA was isolated from six different developmental stages of feverfew flowers (Fig. 2a). Tripure isolation reagent (Roche, Mannheim, Germany) was used for extraction of total RNA according to the manufacture’s protocol. RNA samples were then treated with DNAse I (Invitrogen, USA) and purified. Sequencing was performed using a 454 GS FLX Titanium instrument. The raw reads were assembled after adaptor trimming using Newbler (454 Life Sciences Corporation). BlastX (using the top five hits) against the NCBI database and InterProScan (Jones et al. 2014) were applied to annotate the transcripts, using the top five hits. Illumina reads were mapped against this assembly using bowtie2 with the option–a (report all alignments). Subsequently, eXpress (Roberts & Pachter 2013) was used to estimate expression in the different stages. Finally, DEseq (Anders & Huber 2010) was used to test for differential expression in subsequent stages, based on the est_counts values obtained from eXpress. Here, the three different replicates for each stage were taken into account. DEseq tests for differential expression based on a model using the negative binomial distribution; the FDR threshold for this test was set to 0.05.

To find a set of genes most likely to be relevant for extracellular transport of feverfew sesquiterpene lactones we focused on the contigs which were differentially expressed between at least two subsequent ovary developmental stages. Expression data of feverfew biosynthesis pathway genes together with the rest of selected contigs was imported to the GeneMaths XT (Version 2.12). Log transformation of data in the base of 2 followed by data normalization of complete set with the *average* algorithm to calculate the offset was used for data preparation. Cluster analysis was done on the full-set data by using hierarchical clustering with complete linkage (Supplementary Fig. 1).

### RACE-ready cDNA synthesis and RACE-PCR

Nine candidate TpLTPs selected from RNA-sequencing data analysis. All LTPs were checked for the full length CDS according to the available feverfew sequence information and a RACE-PCR approach was performed where the coding sequence was not complete or the secretion signal was missing. 5′ and 3′ cDNA from feverfew RNA was synthesised according to the manufacturer’s protocol (SMARTer™ RACE cDNA Amplification, Clontech). Gene-Specific Primers for the RACE reaction were designed for the 5′and 3′of 5 LTP candidates. The primers were between 23 and 28 nucleotides long, having a GC ratio of 50–70%, a Tm value above or equal to 70 °C, not complementary to the Universal Primer Mix and a 15 bp overlap with the pRACE vector is added to their 5′ end (unless otherwise mentioned). Touchdown PCR program consisting of 5 cycles at 94 °C for 30 s followed by 72 °C for 3 min, then 5 cycles at 94 °C for 30 s, 70 °C for 30 s, and 72 °C for 3 min, and finally 25 cycles at 94 °C for 30 s, 68 °C for 30 s and 72 °C for 3 min. RACE-PCR products were characterized on an agarose gel and the purified band was cloned into pRACE vector for subsequent sequencing according to manufacturer’s protocol (Clontech, USA).

### Isolation and cloning of candidate feverfew LTPs

After getting the full length sequences of all LTPs Q5 High-Fidelity polymerase PCR was used for full length genes amplification. Feverfew RNA from developmental stage 4 was used to synthesize the cDNA according to manufacturer’s protocol (iScript™ cDNA Synthesis Kit, Bio-Rad). Full length sequences were amplified from feverfew cDNA. Primers are listed in Supplementary Table 1. All TpLTP*s*, except *TpLTP3* were cloned into pCR™8/GW/TOPO® (Invitrogen™) entry vector by the addition of an adenosine overhang into their 3′ end post amplification. An overhang of CACC was added to the forward primer of *TpLTP3* and was cloned into entry vector pENTR™/D-TOPO® (Invitrogen™). TpLTP 1–8 sequences have been deposited in the GeneBank under the accession numbers of MG550248 to MG550255.

### LTPs plasmid construction for in planta expression

pCR™8/GW/TOPO® plasmids except for *TpLTP3* were linearized by HincII or EcoRV-HF (©New England Biolabs). An LR (Gateway® LR Clonase® II Enzyme mix, Invitrogen™) reaction was done for all LTPs to clone individual LTP genes into plant binary vector pB7WG2 (Karimi et al. 2002) between the right and left borders of the T-DNA to express them under the control of cauliflower mosaic virus promoter (p35S) for plant transformation.

### Plasmid construction of *TpLTP1*^*GPI*^ and *TpLTP3*^*∆GPI*^

First *TpLTP*1 was amplified with forward primer 5′CACCATGGAAAACAAAGGAAATAAAATGAC 3′ and reverse primer 5′ CGTCCGATTTCCCTCCTTC 3′ to remove the stop codon. The GPI anchor motif of *TpLTP*3 was then amplified with forward 5′ CACCATGGTTAATTCAAACACCACCATTATG 3′ and reverse primer 5′GCTACCTCCATTTCCAGATGC 3′. *TpLTP3* was amplified with forward 5′ CACCATGGTTAATTCAAACACCACCATTATG 3′ and reverse primer 5′ GAAGTAGTATGCTATGAAGATAGCAAC 3′ to remove the GPI anchor (resulting in *TpLTP3*^∆*GPI*^). Later *TpLTP1* was amplified with forward primer 5′ ATTAGAAGTAGTATGCTATGAAGATAGCAACCGCTAAGCAAATGATAGTCGAAA 3′ and reverse primer 5′ GATCGTGAACTGCATTACTAGATGCTCCATTCGTCCGATTTCCCTCCTTC 3′ to add the GPI anchor to LTP1 (*TpLTp1*^*GPI*^). Later they were transformed into pGWB508 (https://www.ncbi.nlm.nih.gov/nuccore/126145056; https://www.addgene.org/74850/) with an LR reaction to put the fragments between the right and left border of gateway sites of the binary vector.

### Transient expression in *Nicotiana benthamiana* and total metabolites extraction

*Agrobacterium tumefaciens* mediated plant transformation (agro-infiltration) was performed according to the description of van Herpen et al. (2010). Agrobacterium cultures were started from glycerol stocks into LB medium with the addition of kanamycin (50 mgl^−1^) for the pathway genes and spectinomycin (50 mgl^−1^) for LTPs. Rifampicin (37.5 mgl^−1^) was added to all cultures since AGL0 strain has chromosomal resistance to rifampicin (Lazo et al. 1991). Cultures were started in a 5 ml LB media for 1 day which was followed by addition of 5 ml and incubation for one more day at 28 °C. Cells were harvested by centrifugation at 3500×g for 15 min at 20 °C. The bacterial pellet was resuspended by agroinfiltration buffer. The buffer was prepared by adding 10 mM MES buffer and 10 mM MgCl2 supplemented with 0.1 mM acetosyringone (49- hydroxy-39, 59-dimethoxyacetophenone, Sigma). The optical density of cultures were adjusted to 0.5 at the wavelength of 600 nm. After mixing of the cultures for coexpression studies they were incubated on a roller mix for 120–150 min. Mixtures were infiltrated to the 4-week-old *N. benthamiana* plants. Fully developed leaves were infiltrated via a 1 ml syringe without needle to the abaxial part of the *N. benthamiana* leaves which were grown with a day-night temperature of 22–20 °C and a day-night rhythm of 16 h light (starting at 06.00) and 8 h dark (starting from 22.00 Ectopic expression of the LTPs together with the costunolide and parthenolide pathway caused necrotic lesions in leaves at 5 days post-infiltration (dpi). Therefore, leaves were harvested at 3 and 4 dpi for the costunolide and parthenolide pathway, respectively. Harvested leaves were snap-frozen in liquid N_2_. Samples were ground by mortar and pestel. 100 mg of each sample was extracted with 300 µl of methanol. Brief vortexing and 15 min of sonication was followed by 15 min of centrifugation at 13,000×g. 0.45 µm inorganic membrane filters (Minisart® RC4, Sartorius, Germany) was used to filter 200 µl of extract. Samples were kept at − 20 °C until being injected to the UPLC-MRM-MS.

### Apoplast extraction

Agroinfiltrated *N. benthamiana* leaves for costunolide or parthenolide pathway with or without TpLTPs were harvested and were transferred to the lab. Leaves were cut into half from the main vein (by cutting off the main midrib) and also were cut alongside their edges to facilitate the extraction. Half leaves were washed properly to remove cell content leakage due to physical damage and gently dried with a paper towel. Each leaf was weighed and then placed in a beaker containing demineralised water to cover the whole leaf. A vacuum pressure for 20 min was applied to the samples in a desiccator where water penetrated to the extracellular space of leaves by slightly releasing the negative pressure. Then half leaves were gently dried with paper tissue and weighed again. Samples then were placed in a PVC tube containing a sieve at the bottom and which was then placed in a 50 ml Greiner centrifuge tubes (CELLSTAR^R^) and were moderately centrifuged at 400×g for 15 min to avoid cell damage according to (Witzel et al. 2011). Extracted apoplast weight and remaining leaf weight was recorded. Apoplast wash was then centrifuged at 13,000 rpm to get rid of any contaminations from leaf surface and then 100 µl of apoplast was filtered through a 0.45 µm inorganic membrane filter (Minisart® RC4, Sartorius, Germany) and measured by UPLC-MRM-MS.

In order to correct the data based on the vacuum infiltration efficiency and apoplast extraction efficiency the below formulas was used:

Apoplast Volume (µl) = Leaf Weight After Infiltration (µl) − Leaf Weight Before Infiltration (µl).

Absolute (total) Apoplast Content (µg) = Apoplast Volume (µl)/10(µl) (measured sample) × LC MS data apoplast sample.

Extracted Apoplast Content = Liquid Volume/10 (measured sample) × LC MS data apoplast sample.

Apoplast Content still in Leaf = Total Apoplast Content − Extracted Apoplast Content.

Content in Full Leaf = Weight After Centrifuge/100 (extracted sample) × LC MS data leaf sample × 300 (extraction fluid)/10 (measured sample).

Non Apoplastic Content = Content in Full Leaf−Apoplast Content still in Leaf.

Percentage in Apoplast = Total Apoplast Content/Not Apoplast Content × 100.

### UPLC-MRM-MS analysis and quantification of costunolide, parthenolide and their conjugates

Costunolide, parthenolide and their glutathione and cysteine conjugates were measured in a targeted metabolomics analysis by using the Waters Xevo tandem quadrupole mass spectrometer equipped with an electrospray ionization source and coupled to an Acuity UPLC system (Waters) as described by (Liu et al. 2011) with some modifications and optimization. A BEH C18 column (100 × 2.1 mm, 1.7 µm; Waters) was used for chromatographic separation. A UPLC H_2_O:acetonitrile gradient was applied to the column starting from 5% (v:v) acetonitrile in H2O for 1.25 min and rising to 50% in 2.35 min. the gradient was increased to 90% (v:v) acetonitrile in H2O in 3.65 min. this gradient was kept for 0.75 min before returning to 5% acetonitrile by using a 9 s gradient. The column was equilibrated by using the same solvent composition for 1.85 min. column temperature was kept at 50 °C with a flow rate of 0.5 ml min^−1^. The capillary voltage was 3.0 kV and source and desolvation temperatures were 150 and 650 °C, respectively. 10 µl of each sample was used for injection. Desolvation gas and cone flow were set at 50 and 1000 l h^−1^, respectively. Cone voltage for detected compounds was optimized by the Waters IntelliStart MS Console. Collision-induced dissociation for fragmentation was done by using Argon in the ScanWave collision cell. For identification and quantification of parthenolide, costunolide and their conjugates Multiple Random Monitoring (MRM) was used. MRM transitions for costunolide was [M + H]^+^ = 145; [M + H]^+^ = 145 and [M + H]^+^ = 215.09 and for costunolide-Cys was the same for conjugates as well. MRM transitions for parthenolide were [M + H]^+^ = 144.9; [M + H]^+^ = 185.224 and [M + H]^+^ = 231.4. Parthenolide-Cys transitions were set at [M + H]^+^ = 158.97; [M + H]^+^ = 187.12 and [M + H]^+^ = 231.01 and the transitions for parthenolide-GSH were set at [M + H]^+^ = 185.224 and [M + H]^+^ = 231.229. Mass spectrometer was operated in positive electrospray ionization mode for all above mentioned compounds.

### LC-Orbitrap-FTMS analysis of 3β-hydroxyparthenolide and 3β-hydroxyparthenolide-conjugates

A methanolic:formic acid (1:1000) extract of *N. benthamiana* leaves was injected to LC-Orbitrap-FTMS (Thermo Scientific) consisting of an Accela HPLC, an Accella photodiode detector (array) and coupled to a LTQ/Orbitrap hybrid mass spectrometer, connected to ESI source. An analytical column (Luna 3 µ C18/2 100A; 2.0 × 150 mm; Phenomenex, USA) was used for chromatographic separation. Eluent A (degassed) [HPLC grade water: formic acid (1000:1, v/v)] and eluent B [acetonitrile: formic acid (1000:1, v/v)] were used and the flow rate was set to be 0.19 ml min^−1^. A gradient of 5 to 75% acetonitrile in 45 min was applied which was then followed by 15 min wash and layer equilibration. Full scan mass analysis (FTMS) were recorded at resolution of 60000 and the resolution for MS^n^ scans was set to 15000. Calibration of FTMS was done externally in negative ionisation mode by using sodium formate clusters in the range m/z 150–1200, and automatic tuning was performed on m/z 384.93. Injection volume for each sample was 5 µl.

### Statistical analysis

The experiments were conducted based on completely randomized design (CRD). Each treatment was replicated four times. Data was analysed with ANOVA followed by means comparison. Differences among mean values were compared using Duncan’s Multiple Range Test at the probability level of 0.05 (P ≤ 0.05).

## Results

### Identification of candidate lipid transfer protein genes from co-expression analysis

Based on our experience with the LTPs that play a role in artemisinin biosynthesis in *A. annua* (Wang et al. 2016), we searched for candidate feverfew LTPs that may be active in the costunolide/parthenolide biosynthesis pathway. First, a feverfew trichrome enriched reference transcriptome assembly was obtained. The number of contigs longer than 500 bp was 29,799 and the total contig length 27 Mb. Subsequently, the expression profile over six ovary developmental stages was obtained from triplicate cDNA samples per developmental stage. RNA-seq reads were mapped to the reference transcriptome assembly. On average, 35 M ± 10 M reads per developmental stage were obtained, and the average alignment rate was 79% ± 4%. Analysis of the expression profiles indicated that 5414 contigs were differentially expressed between at least two subsequent stages. To further narrow down this set to the most relevant genes, BLAST was used to identify the known costunolide/parthenolide pathway genes as well as sequences with similarity to LTPs, P450s and ATP binding cassette membrane transporters. LTP sequences were selected based on GO terms associated with the BLAST hits and the conserved 8 cysteine motif of NsLTPs (Kader 1996; Supplementary Fig. 4a). This resulted in a set consisting of 13 putative LTP, 75 putative P450, and 35 putative ABC transporter sequences (Supplementary Fig. 1).

The expression profile of the known costunolide/parthenolide biosynthesis genes (*GAS*, *GAO*, *COS*, and *PTS*) was used to identify those *TpLTP* genes that show a similar expression profile as the sesquiterpene pathway genes, resulting in selection of eight candidate LTP sequences (Fig. 2). *LTP6* and *7* were clustered together while *LTP1* to *LTP5* were closely clustered together with pathway genes. *LTP8* was in a separate branch in the cluster analysis. For these eight candidate *TpLTP*s, the coding sequence was cloned into a binary expression vector under the control of the CaMV 35S promoter. The LTPs were functionally analysed by transient co-expression with the costunolide or parthenolide biosynthesis pathway genes in *N. benthamiana*.

### The effect of TpLTPs on costunolide and parthenolide levels

**Fig. 3:**
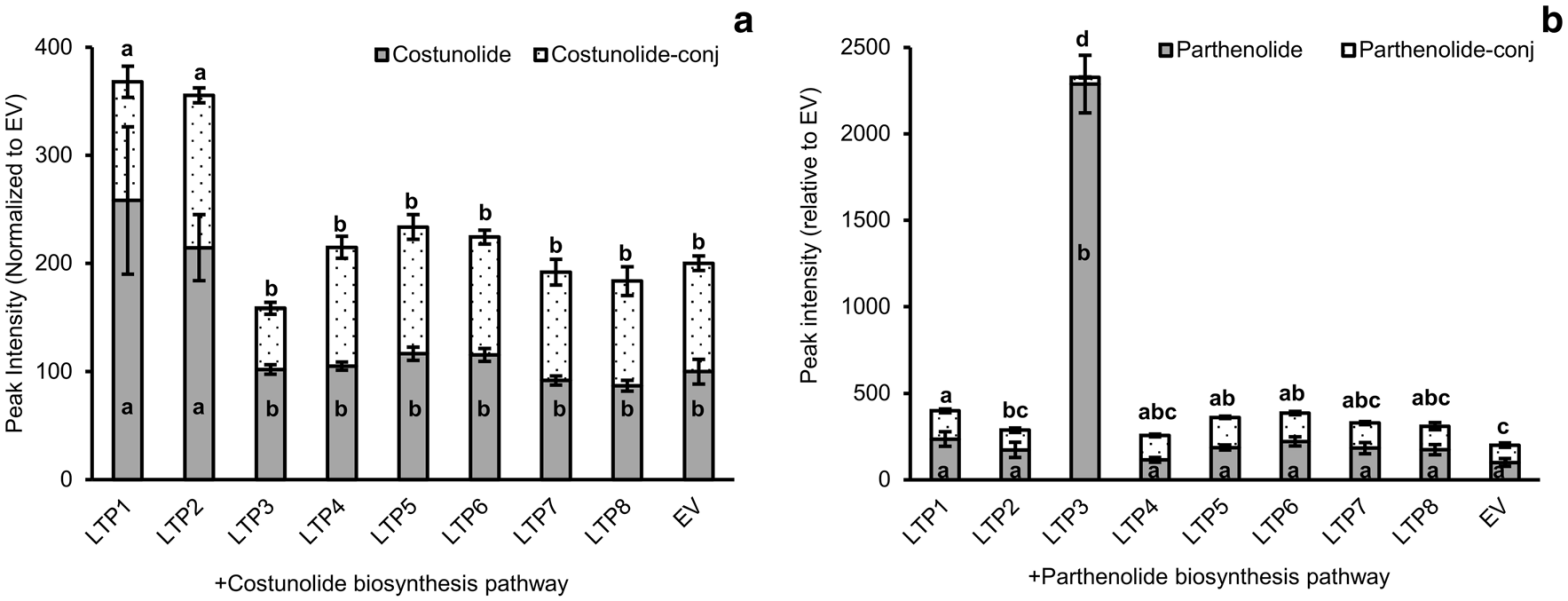
Quantification of the effect of individual feverfew Lipid Transfer Proteins (LTPs) on metabolite accumulation as analysed by UPLC-MRM-MS. **a** TpLTP effect on the accumulation of costunolide biosynthesis pathway products at 3 dpi. **b** *Tp*LTP effect on the accumulation of parthenolide biosynthesis pathway products at 4 dpi. All values are normalized to the levels produced by the costunolide and parthenolide biosynthesis pathways in combination with an empty vector (EV) (n = 4). Means followed by a different letter are significantly different (P ≤ 0.05)

In order to test whether any of the eight TpLTPs has an effect on costunolide production, the costunolide pathway genes (*AtHMGR*, *TpGAS1.5*, *CiGAO*, *CiCOS*) were co-expressed with each individual TpLTP in 4-week-old *N. benthamiana* leaves by co-infiltration of *Agrobacterium* batches carrying the different expression constructs. The relative dosage of the costunolide pathway genes was kept equal between experiments by using empty vectors (EV) where needed and a *p19* construct was used to repress the silencing mechanisms of *N. benthamiana* (Voinnet et al. 2003). After harvest, metabolites were extracted for targeted metabolite analysis using UPLC-triple quadrupole-MS. The levels of free costunolide and costunolide conjugated to sugar molecules in leaves expressing the costunolide pathway + EV were set at 100%. Figure 3a shows that only co-expression of TpLTP1 and TpLTP2 results in a substantial increase in both free and conjugated costunolide (2.4 and 2.65 times, respectively; Fig. 3a). Of the other LTPs, only TpLTP3 had a significant effect, slightly decreasing the level of conjugated costunolide (Fig. 3a).

Subsequently, the eight TpLTPs were tested for their effect on parthenolide pathway product accumulation. For this, the parthenolide pathway genes (*AtHMGR*, *TpGAS1.5*, *CiGAO*, *CiCOS*, *TpPTS*) were co-expressed with each of the eight individual TpLTPs in 4-week-old *N. benthamiana* leaves by agro-infiltration of the expression constructs. The level of free parthenolide and parthenolide conjugates in leaves expressing the parthenolide pathway + EV were again set at 100%. In contrast to their effect on costunolide, neither TpLTP1 nor TpLTP2 had an effect on free or conjugated parthenolide levels (Fig. 3b). However, expression of TpLTP3 with the parthenolide biosynthesis pathway genes resulted in a remarkable 23-fold increase in free parthenolide, while the level of conjugated parthenolide decreased (Fig. 3b). Thus, three of the eight TpLTPs that showed co-expression with the biosynthetic pathway genes (Fig. 2) have an effect on the accumulation of sesquiterpenes, with TpLTP1 and TpLTP2 showing specificity for costunolide and TpLTP3 for parthenolide.

Analysis of the free and conjugated costunolide levels in the parthenolide-pathway experiment, shows that TpLTP1 and TpLTP2 are still active in enhancing accumulation of free costunolide, but they do not contribute to accumulation of free or conjugated parthenolide (Supplementary Fig. 2)**.** This indicates that the enhanced costunolide accumulation in the presences of TpLTP1 or TpLTP2 is not available for TpPTS activity. This would be consistent with the LTPs mainly having an effect on product accumulation in the apoplast of the *N. benthamiana* leaves, where costunolide is no longer available for TpPTS. Therefore, we next analysed the effect of TpLTP1 to 3 on costunolide and parthenolide pathway product accumulation in the apoplast.

### Expression of TpLTP1 to 3 results in increased levels of costunolide and parthenolide in the apoplast

**Fig. 4:**
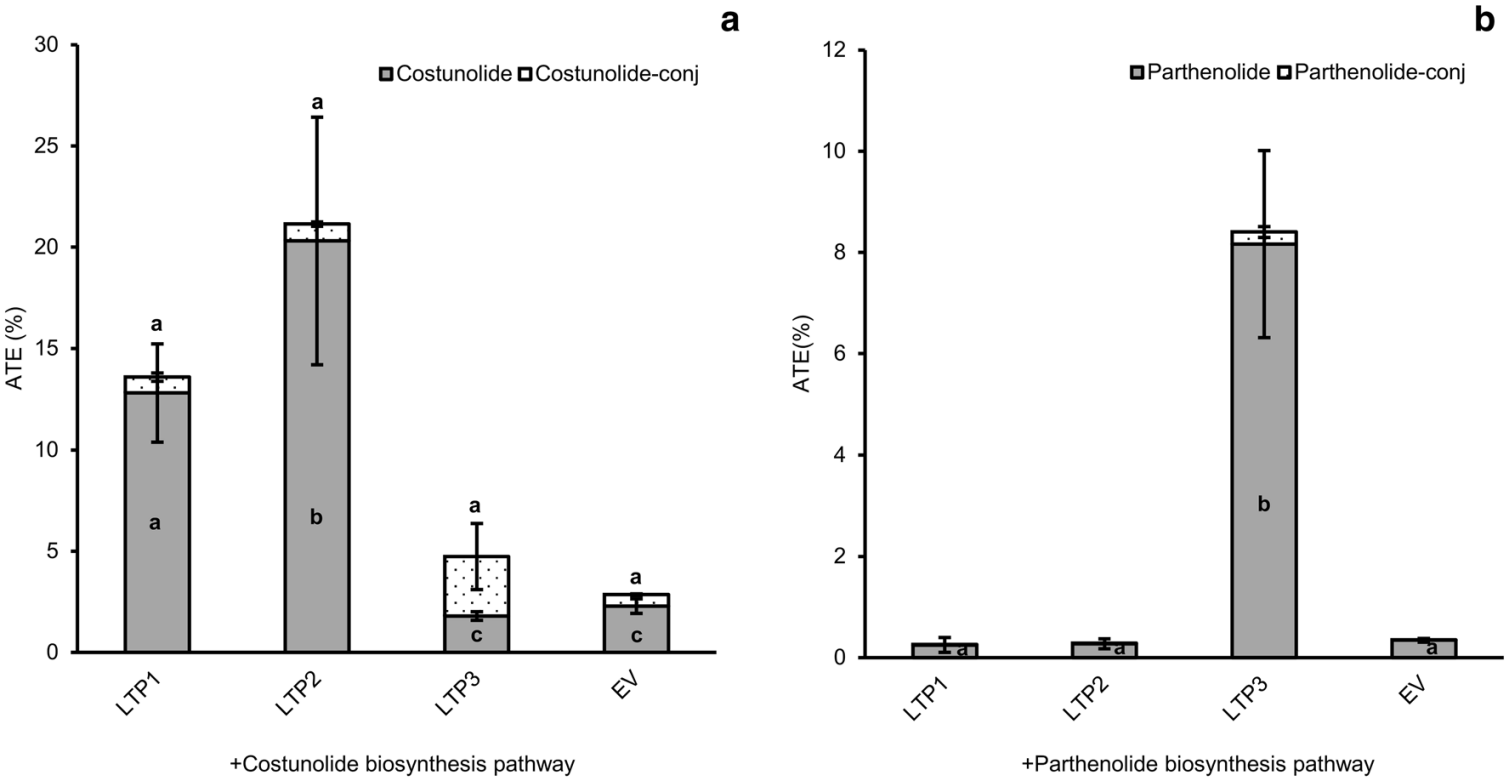
Quantification of the effect of TpLTPs on metabolite accumulation in the apoplast as analysed by UPLC-MRM-MS. **a** Costunolide (ATE_costunolide_) and costunolide-conjugate apoplast transport efficiency; grey bars represent free costunolide and black-dotted bars represent sum of costunolide-conj. **b** ATE_parthenolide_ and ATE_parthenolide-conj_; grey bars represent free parthenolide and black-dotted bars represent the sum of parthenolide-conj. All values are normalized to yield in (costunolide/parthenolide) pathway + EV (empty vector) (n = 4). Means followed by a different letter are significantly different (P ≤ 0.05)

In feverfew flowers, the costunolide level peaks at stage 3 of flower development, while the parthenolide level peaks at stage 5 of flower development (Fig. 2a). Also the expression profile of *TpLTP1* and *TpLTP2* shows an earlier peak (stage 2) than *TpLTP3* (stage 3) (Fig. 2b). Moreover, more than 90% of the parthenolide and costunolide in feverfew is present in the extracellular space of the glandular trichomes (Liu et al. 2014), indicating an efficient export of these sesquiterpenes from the cell into the apoplast. To test whether the three active TpLTPs boost pathway product accumulation in the apoplast, the apoplast of *N. benthamiana* leaves transiently expressing the pathway-LTP combinations was extracted and analysed. Leaves with transient expression of either the costunolide or parthenolide pathway in absence or presence of the individual TpLTPs were harvested at 4 dpi, briefly vacuum infiltrated with water, followed by slow centrifugation for non-destructive extraction of infiltrated water from the extracellular space. In general, approximately 75% of the infiltrated water was retrieved after centrifugation. After extraction of the apoplast wash fluid, leaves were extracted with MeOH for analysis of the remaining, non-apoplastic, products. Apoplastic wash fluid and leaf extracts were analysed by UPLC-MRM-MS for quantification of free and conjugated compounds. From these data the ratio of compound level in the apoplast of one leaf and the level in the remaining leaf material (with correction for non-extracted apoplast fluid) was calculated. We consider the ratio of ‘apoplast content/leaf content’ to be a measure for overall Apoplast Transport Efficiency [ATE = 100 × (apoplast content/leaf content)]. The data show that also without co-expression of TpLTPs, both free and conjugated costunolide are detected in the apoplast, indicating an intrinsic transport activity for costunolide in *N. benthamiana*. The presence of conjugated costunolide in the apoplast can either be from export of conjugated costunolide or from costunolide conjugating activity (to GSH) in the apoplast of *N. benthamiana* leaves. Without TpLTPs approximately 2% of free and conjugated forms of costunolide are in the apoplast (ATE_costunolide+_ATE_costunolide-conj_ = 4), while with TpLTP1 ~12% and with TpLTP2 ~20% of free costunolide is in the apoplast (Fig. 4a). The effect of TpLTP1 and 2 on free costunolide in the apoplast seems to be specific, because they had no significant effect on sequestration of costunolide conjugates in the apoplast (P < 0.05) (Fig. 4a), while TpLTP3 has no or even inhibitory effect on the accumulation of free and conjugated costunolide (Fig. 3a), TpLTP3 seems to enhance accumulation of conjugated costunolide in the apoplast (ATE_costunolide_ increases from ~0.5 to ~3) which is not significantly different to the EV (Fig. 4a).

Similarly, the ATE_parthenolide_ was determined for leaves expressing the parthenolide pathway genes with or without the TpLTPs. Results show that without TpLTPs, ~0.2% of free parthenolide is in the apoplast (ATE_parthenolide_ = 0.2). TpLTP1 and 2 had no effect on ATE_parthenolide_, but TpLTP3 increased ATE_parthenolide_ from 0.2 to 8%. Again this effect seems to be specific as TpLTP3 did not enhance the level of conjugated parthenolide in the apoplast (Fig. 4b). When co-expressed with the parthenolide pathway, both TpLTP1 and 2 enhanced the level of free costunolide in the apoplast (ATE_costunolide_ increases from 0.2 to 12 and 14, respectively) (Supplementary Fig. 3). Overall, the results show that TpLTP1 and TpLTP2 are more active in apoplast accumulation of costunolide, while TpLTP3 is more active in apoplast accumulation of parthenolide.

### Parthenolide accumulation not significantly increased by TpLTP1/2, but costunolide accumulation reduced upon co-expression of TpLTP1/2/3

**Fig. 5:**
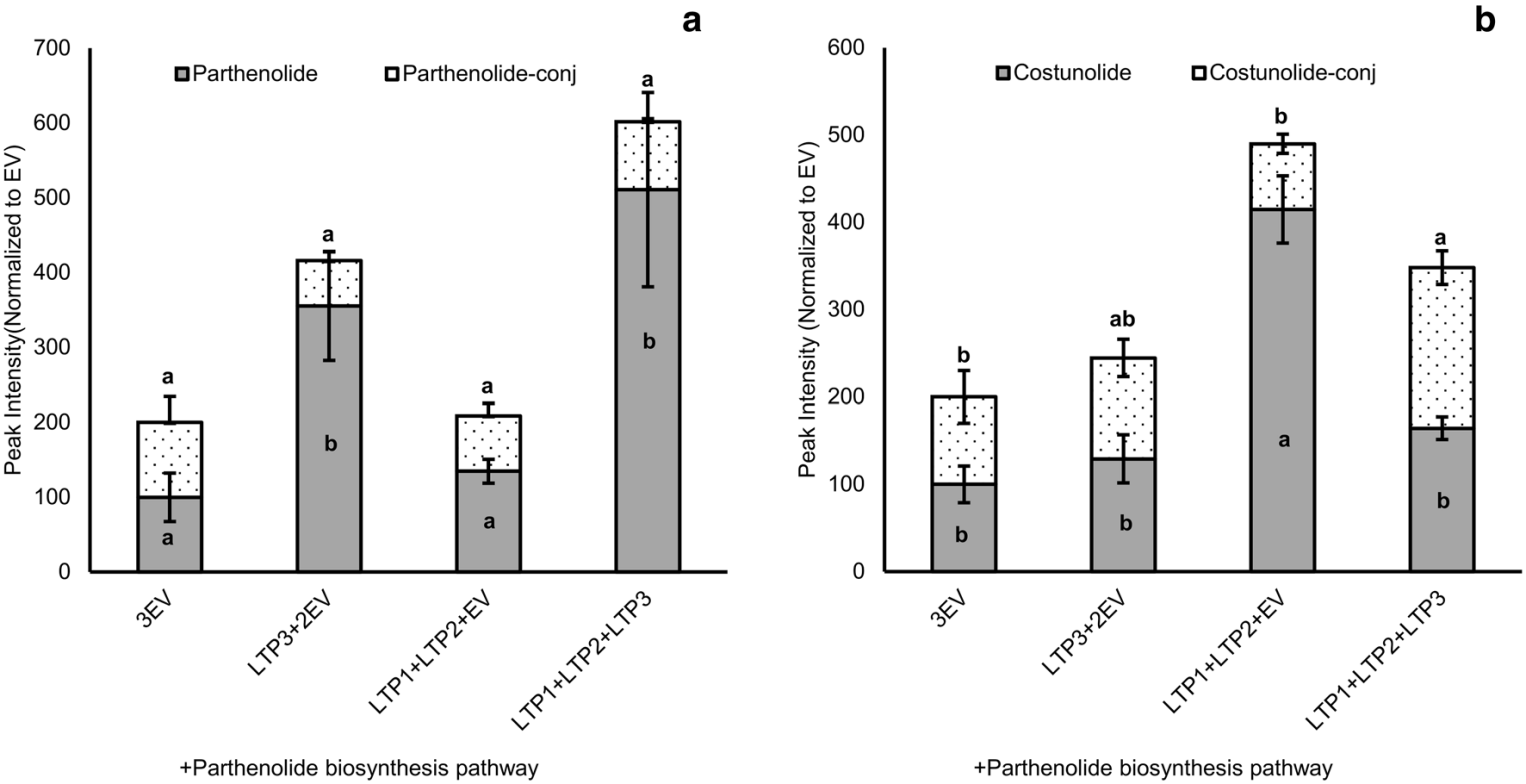
UPLC-MRM-MS quantification of products of the parthenolide pathway transiently expressed in combination with TpLTPs in *N. benthamiana*. **a** Level of parthenolide (grey bars) and parthenolide conjugates (black-dotted bars) in leaves expressing the parthenolide biosynthesis pathway genes (*HMGR, GAS, GAO, COS, PTS*) co-expressed with different TpLTPs **b** level of costunolide and costunolide conjugates in leaves expressing the parthenolide biosynthesis pathway with different TpLTPs. (n = 4). Means followed by a different letter are significantly different (P ≤ 0.05)

In feverfew the three LTPs are presumably expressed together at the same time and result in extracellular accumulation of costunolide, but mostly parthenolide. Therefore, we performed an experiment in *N. benthamiana* where all three functional LTPs were coexpressed with the parthenolide pathway. The data show that there is no significant difference between TpLTP3 + 2EV and TpLTP3 + TPLTP2 + TpLTP1 for free parthenolide production (Fig. 5a). Analysis of costunolide and its conjugates showed that TpLTP3 did not contribute to free costunolide accumulation, while co-expression of TpLTP1 and 2 significantly enhanced free costunolide accumulation. Co-expression of TpLTP3 with TpLTP1 and 2 ruled out the positive effect of the latter two LTPs (Fig. 5b). We note that accumulation of parthenolide in these experiments did not reach to the same level as for the experiment shown in Fig. 3. This may be explained by a dilution effect on the parthenolide biosynthesis pathway gene dosage. In the experiment of Fig. 3 the *PTS* expression construct was co-infiltrated with six other expression constructs, while in the experiment of Fig. 5, the *PTS* expression construct was co-infiltrated with eight other constructs. At a high number of co-infiltrated expression constructs not all cells of the infiltrated leaf may be transformed by all gene constructs, which would reduce overall parthenolide biosynthesis and LTP activity.

### *Tp*LTPs prevent influx of costunolide and parthenolide into leaf cells

**Fig. 6:**
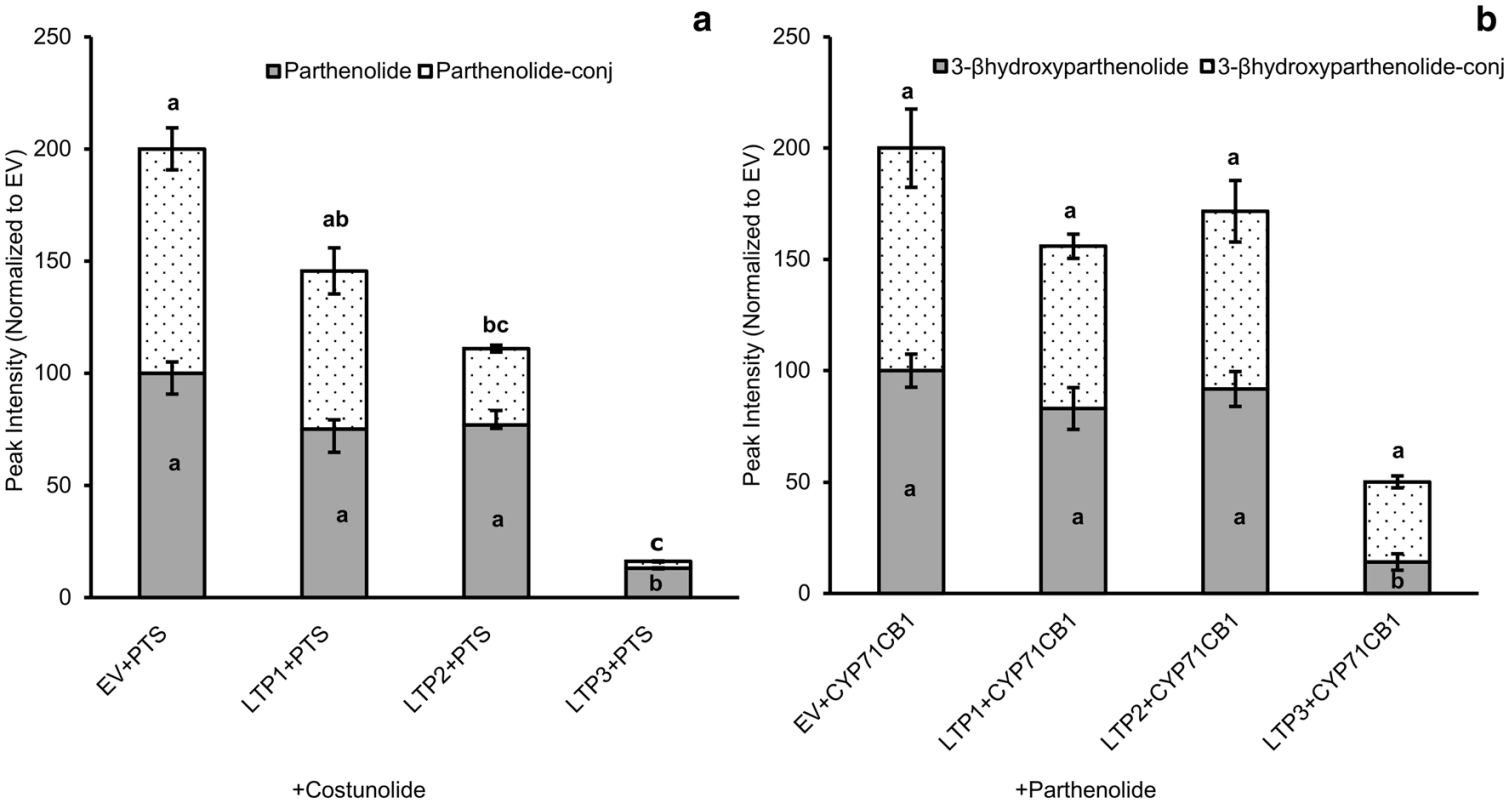
Intercellular bioconversion of costunolide to parthenolide by parthenolide synthase and parthenolide to 3β-hydroxyparthenolide by 3β-parthenolide hydroxylase in *N. benthamiana* leaves. Parthenolide synthase or 3β-costunolide hydroxylase were infiltrated into *N. benthamiana* leaves together with different LTPs. After 3 days, substrates (either costunolide or parthenolide) were vacuum infiltrated into leaf disks of 1 cm diameter. EV was used as a control. Metabolites were extracted with methanol and analysed with LC–MS. **a** UPLC-MRM-MS analysis of extracted metabolites from leaves expressing parthenolide synthase, vacuum infiltrated with costunolide and incubated for 120 min. **b** Bioconversion of parthenolide to 3β-hydroxyparthenolide. Apoplast vacuum infiltration of parthenolide and extraction of leaf metabolites at 120 min (analysed by LC-Orbitrap-FTMS). Means followed by a different letter are significantly different (P ≤ 0.05)

An alternative way to test for LTP activity and specificity *in planta* is by a substrate exclusion assay (Wang et al. 2016). In such an assay, a substrate is infiltrated into the apoplast of leaves and the uptake of the substrate by the cells is estimated by analysis of the conversion of the substrate inside the cells. If ectopic expression of an LTP (partly) prevents this bioconversion, that suggests that the LTP helps to retain the compound in the apoplastic space outside the cellular PM. For monitoring the influx of infiltrated costunolide into cells we expressed *Tp*PTS in leaves of *N. benthamiana* and used parthenolide production as a measure for the influx of costunolide. *PTS,* in combination with an empty vector or a TpLTP were expressed in *N. benthamiana* leaves and at 3 dpi leaf discs were taken from the agro-infiltrated leaves. The leaf discs were vacuum infiltrated with the substrate costunolide, were harvested and rinsed at 2 h post substrate infiltration and were subsequently analysed for costunolide and parthenolide. In leaves expressing *PTS* and *EV* and infiltrated with costunolide, both parthenolide and parthenolide conjugates were detected, indicating efficient influx of costunolide into the cells and bioconversion by the ectopically expressed parthenolide synthase (Fig. 6a). Co-expression of TpLTP1 or TpLTP2 with PTS reduced parthenolide production by approximately 20% (Fig. 6a). In contrast, co-expression of *TpLTP3* with *PTS* blocked bioconversion of costunolide to parthenolide by almost 90% (Fig. 6a), indicating efficient costunolide exclusion from the cells by TpLTP3. In a similar exclusion assay with parthenolide as infiltrated substrate, the ectopic expression of a parthenolide 3β-parthenolide hydroxylase [CYP71CB1 (Liu et al. 2014)] was used to monitor bioconversion of parthenolide to 3β-hydroxyparthenolide and 3β-hydroxyparthenolide conjugates (Fig. 6b). In this exclusion assay, the co-expression of either TpLTP1 or TpLTP2 with CYP71CB1 could block parthenolide influx by 18% or 9%, respectively (Fig. 6b), while co-expression of TpLTP3 with CYP71CB1 blocks influx of parthenolide by 86%. Thus, in the substrate exclusion assays with the three LTPs, only TpLTP3 efficiently blocks both costunolide and parthenolide influx into the cell (Fig. 6a, b).

### TpLTP3 has a GPI anchor domain which is essential for its activity

**Fig. 7:**
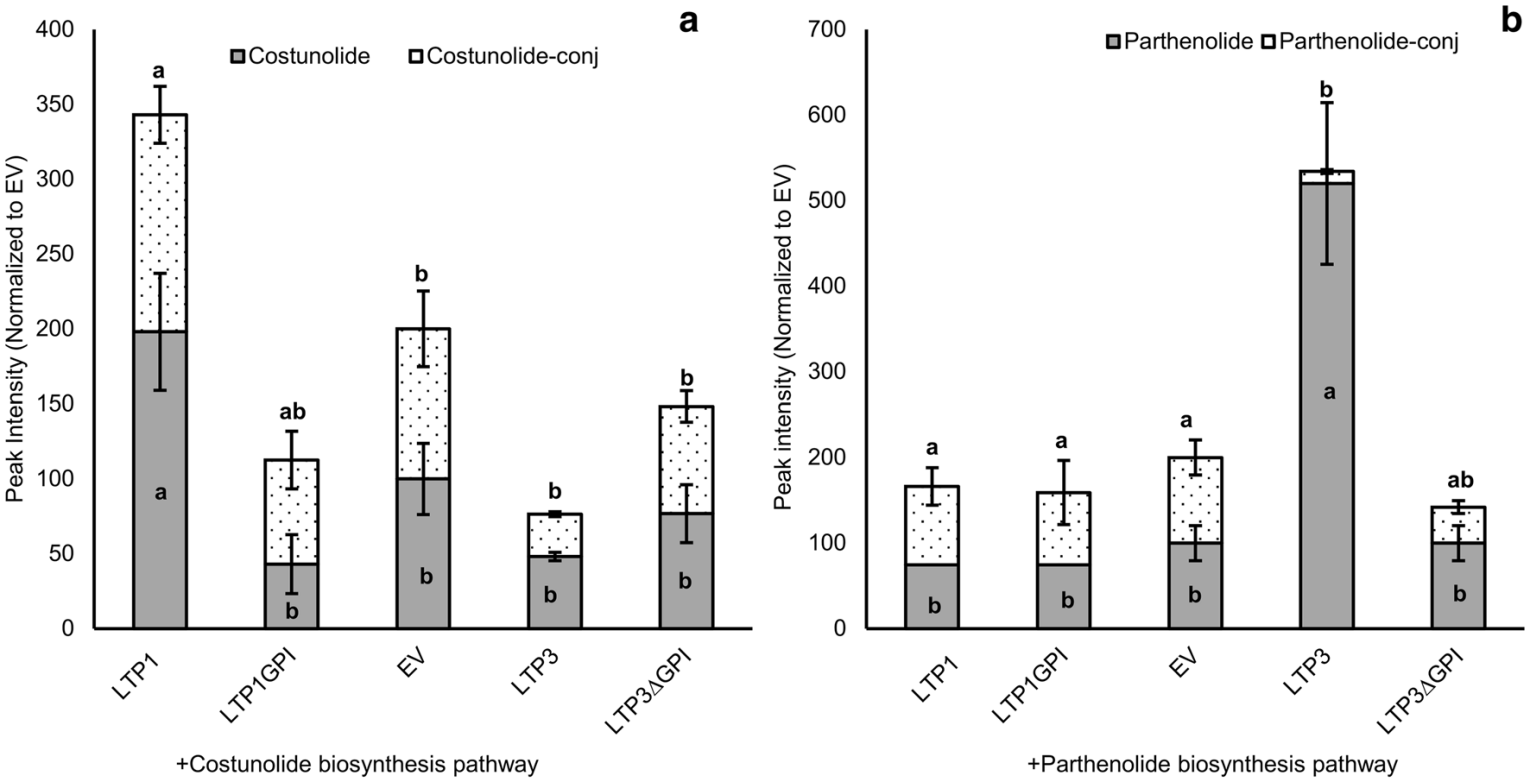
Effect of addition of a GPI anchor to LTP1 and truncation of the GPI anchor from LTP3 on metabolite accumulation in *N. benthamiana*, as analysed by UPLC-MRM-MS. **a** effect of the addition of a GPI anchor to LTP1 and removal of the GPI anchor from LTP3 on the production of costunolide (grey bars) and costunolide conjugates (black-dotted bars) when transiently coexpressed with the costunolide biosynthesis pathway genes and **b** effect of addition of GPI anchor to LTP1 and removal of LTP3 GPI anchor on the production of parthenolide (grey bars) and parthenolide conjugates (black-dotted bars) upon transient coexpression with the parthenolide biosynthesis pathway genes (n = 4). Means followed by a different letter are significantly different (P ≤ 0.05)

The amino acid coding sequence of TpLTP3 indicates that it contains an extended domain with a structural motif that is associated with addition of a GPI-anchor. TpLTP3 therefore belongs to the LTPG (GPI-anchored) class of LTPs. In Arabidopsis and cotton, LTPG proteins are involved in the deposition of leaf cuticular waxes and transport of phosphatidylinositol monophosphates in fibres, respectively (Kim et al. 2012; Deng et al. 2016). The role of the GPI anchor domain in these processes is unknown. In order to identify the role of the GPI-anchor domain of TpLTP3 we produced a truncated version of TpLTP3 by removing the GPI anchor site (TpLTP3^∆GPI^) (Supplementary Fig. 4b). In addition, we tested whether the GPI anchor domain of TpLTP3 enhances the functionality of TpLTP1 by linking it to the C terminus of TpLTP1 (TpLTP1^GPI^). Co-expression of TpLTP3^∆GPI^ with either the parthenolide or costunolide biosynthesis pathway showed that the truncated protein no longer increases parthenolide accumulation (Fig. 7a). The deletion of the anchor motif did not affect costunolide production. The addition of the GPI anchor motif to TpLTP1 resulted in a loss of TpLTP1 activity in costunolide accumulation (Fig. 7a). Addition of the GPI anchor to TpLTP1 did not have a positive effect on accumulation of parthenolide (Fig. 7b). The GPI anchor domain therefore seems to be essential for the function of TpLTP3, but does not provide parthenolide transport activity to TpLTP1. Moreover, adding the anchor compromises the activity of TpLTP1 in costunolide production.

## Discussion

### Specific boosting of sesquiterpene accumulation by individual feverfew LTPs

Here we discovered that three feverfew trichome LTPs are specific for different sesquiterpene lactones stored in the trichomes. This was assessed by characterizing the efficiency of the three feverfew LTPs in increasing product formation of a transiently reconstituted costunolide and parthenolide biosynthesis pathway in *N. benthamiana* leaves. The increase in free costunolide production by coexpression of TpLTP1 and TpLTP2 with the costunolide pathway genes (Fig. 3a) seems to be caused by the enhanced accumulation of free costunolide in the apoplast (Fig. 4a). In contrast, coexpression of TpLTP3 had no effect on costunolide accumulation when co-expressed with either the costunolide or parthenolide pathway genes (Figs. 3b, 4b). However, TpLTP3 had a big effect on free parthenolide production, which, again, can be ascribed to enhanced accumulation into the apoplast (Fig. 4b). Combined, these results show that these LTPs play a role in transport of costunolide (TpLTP1/2) and parthenolide (TpLTP3) to the apoplast. We interpret these results as a transport activity over the cell wall, although we do not have detailed spatial resolution of the extracellular product accumulation.

The substrate exclusion assays reveal another activity of these LTPs. The results show that the exclusion activity by TpLTP1/2 is limited, while TpLTP3 can effectively exclude influx of both costunolide and parthenolide from the apoplast into the cell. We interpret this result as a transport of general lipid molecules (e.g. waxes, fats, etc.) by these LTPs to the outer cell wall of mesophyll cells of the *N. benthamiana* leaves, thus forming a lipid layer that blocks influx of either costunolide or parthenolide. If this is true, then all three TpLTPs are non-specific LTPs as they act on the sesquiterpene lactones as well as other lipids (Liu et al. 2015; Salminen et al. 2016). We did not test for lipids binding to these LTPs as direct binding studies with sesquiterpene molecules or lipid molecules to each feverfew LTP is beyond this study. Future studies may need to investigate whether interaction of a sesquiterpenes with LTP protein is based on co-binding with a lipid molecule.

### Feverfew TpLTP activity co-dependent on membrane transporters?

For the *Aa*LTP3 from *Artemisia annua*, the activity on (DH)AA depends on co-expression with the membrane transporter *Aa*PDR2 which transports (DH)AA over the PM^10^. In the experiments described in the present study, the activities of TpLTP1/2 and TpLTP3, are found in a background of an existing, low, intrinsic export activity by *N. benthamiana* cells (see Fig. 4a, b EV controls). It could be that the action of the five feverfew LTPs that show little or no activity on costunolide or parthenolide sequestration may be more dependent on co-expression with specific feverfew trichome membrane transporter proteins for these compounds. Alternatively, or in addition, these TpLTPs may have affinity for other products of the feverfew sesquiterpene biosynthesis pathway. Extracts from feverfew trichomes contain an array of different derivatives from costunolide and parthenolide [e.g. costunolide di-epoxide, 3β-hydroxycostunolide, 3β-hydroxyparthenolide, artecanin, artemorin, etc. (Fischedick et al. 2012)] and it will be of interest to determine the activity of the different TpLTPs in combination with membrane transporters expressed in feverfew trichomes for these other feverfew pathway products.

### GPI anchor required for TpLTP3 activity

The mechanism by which LTPs (TpLTP1/2, AtLTP1/3, AaLTP3) enhance the sequestration of sesquiterpenoids in the apoplast is easiest explained when we assume a carrier function over the cell wall. It is therefore surprising that the most active TpLTP in sesquiterpene sequestration belongs to the subclass of GPI-anchored LTPs. The group of LTPs with GPI anchor (NsLTPGs) is a subfamily of the Non-specific LTPs (NsLTPs), and have been shown to function in sporopollenin, suberin, and wax deposition and transport of phosphatidylinositol mono-phosphates (PtdIns3P, PtdIns4P and PtdIns5P) (Edstam et al. 2013; Deng et al. 2016). TpLTP3 is therefore the first member of the nsLTPG subfamily that seems to function in sesquiterpenoid transport. In many instances nsLTPGs function in deposition of lipophilic material on the outside of the cell wall, but in none of these cases it is clear how the supposedly restricted movement of these LTPGs can cause transport over the cell wall. One option may be shedding of the GPI anchor via phospholipase action. However, for LTPGs this seems to more an exception than the rule (Ferguson et al. 2009).

Moreover, we show that TpLTP3 loses its bioactivity without GPI anchor domain, while addition of this domain to TpLTP1 destroys TpLTP1 function. Another option is that GPI-anchored LTPs function as shuttle between membrane transporters and other LTPs that can move freely over the cell wall. However, there is no experimental evidence for such cooperation between different types of LTPs. In theory, a GPI anchor on TpLTP3 only allows for lateral movement in the membrane. However, studies on an LTPG from *Arabidopsis thaliana* show that LTPGs may accumulate in the apoplastic space when expressed early during development (DeBono et al. 2009). Interestingly TpLTP3 displays relatively early expression. Although parthenolide accumulation and TpPTS expression reach to the maximum level at a later stage, parthenolide biosynthesis and accumulation start early in flower development. The GPI anchor prevents free movement over the cell wall, except near structures that penetrate the cell wall like the plasmodesmata (PD). It is therefore intriguing that recently it was shown that GPI anchors are involved in targeting proteins to PD (Zavaliev et al. 2016).

### Wider implications for role LTPs in cell-to-cell transport of lipophilic compounds?

It has now been demonstrated that for hydrophobic sesquiterpenoids, specific LTPs may enhance their transfer over a hydrophilic plant cell wall. We speculate that facilitated transfer over the cell wall may be relevant for other lipophilic compounds as well. Table S1 lists the predicted LogD (Leo et al. 1971) of different plant hormones. The table shows that the LogD of some strigolactones is close to that of the sesquiterpenoids in the present study. In petunia it has been shown that an ABCG-type membrane transporters (*Ph*PDR1) is involved in secretion of strigolactones. Moreover, the apical subcellular localisation of *Ph*PDR in root cells suggests that strigolactones may be subject to polar apical transport in the root tip (Sasse et al. 2015). Such polar transport would require transfer of the strigolactones over two cell walls and this raises the question whether the transfer over the cell walls is facilitated by LTPs.

It can not be excluded that the genetic background and intrinsic membrane transport capacity by membrane transporters of *N. benthamiana* affects the outcome of the functional assay of the different LTPs from Feverfew. For instance, when intrinsic membrane transporters from *N. benthamiana* cannot act on ectopically expressed pathway intermediates, the ectopic expression of a heterologous LTP may not be effective. Indeed, the action of Artemisia AaLTP3 in accumulation of artemisinin in transient expression assays was enhanced when co-expressed with an ABC membrane transporter from *A. annua* (Wang et al. 2016). Future research will have to show if optimal combinations between specific membrane transporters and LTPs can reveal effective combinations that may be used to enhance transfer of specific lipophilic compounds over plant cell walls. Potentially, such dual transient expression assays may be required to reveal the function of other LTPs. Our results indicated that TpLTP1 and TpLTP3 are good candidates for a role in apoplastic transport of sesquiterpene lactones in Feverfew. However, final validation may have to come from stable transformation of *T. parthenium* aimed at silencing of the endogenous genes. Testing only overexpression of these LTPs for effect on apoplastic accumulation of specific compounds in Feverfew may be inconclusive when the high endogenous level of these LTPs is already saturating for apoplastic transport activity, or when activity of additional higher levels of LTPs is limited by normal levels of membrane transporter activity. Future research will have to show if optimal combinations between specific membrane transporters and LTPs can enhance transfer of specific lipophilic compounds over plant cell walls, whether this can be used to engineer extracellular accumulation of desired secondary metabolites and whether this has a role in homeostasis of strigolactones and other plant hormones.

## Supplementary Information

Below is the link to the electronic supplementary material.

Supplementary file1 (DOCX 1963 kb)

## Data Availability

All the data material are deposited in in-house databases and are available on request from the corresponding author.

## Code availability

Not available.
