# Supplementary material for: Individual lipid transfer proteins from Tanacetum parthenium show different specificity for extracellular accumulation of sesquiterpenes
Source: Plant Mol Biol. 2022 Oct 18;111(1-2):153–66. doi: 10.1007/s11103-022-01316-2 (PMC9849177; doi:10.1007/s11103-022-01316-2)
Supplement: Supplementary file 1 — Supplementary file1 (DOCX 1963 kb) [file 11103_2022_1316_MOESM1_ESM.docx]

**Supplementary information**

***Tanacetum parthenium*trichome nsLTPs selected from *in-planta*** **extracellular transport**-**assays, boost extracellular accumulation of parthenolide**

***Arman Beyraghdar Kashkooli^1,4^, Aalt D.J. van Dijk^2^, Harro Bouwmeester^1,3^, Alexander van der Krol^1^****

1. Laboratory of Plant Physiology, Wageningen University and Research, Droevendaalsesteeg 1, 6708 PB Wageningen, The Netherlands.
2. Applied Bioinformatics, Bioscience, Plant Sciences Group, Wageningen University & Research, Wageningen,The Netherlands.
3. Current address: University of Amsterdam, Swammerdam Institute for Life Sciences, Plant Hormone Biology group, Science Park 904, 1098 XH Amsterdam, The Netherlands
4. Current address: Department of Horticultural Science, Faculty of Agriculture, Tarbiat Modares University, PO Box 14115-336, Tehran, Iran.

Orcid IDs: Arman Beyraghdar Kashkooli (0000-0002-4029-9671), Aalt D.J. van Dijk (0000-0002-8872-5123), Harro Bouwmeester (0000-0003-0907-2732), Alexander van der Krol (0000-0001-6585-7572)

^*^: Correspondence: [sander.vanderkrol@wur.nl](mailto:sander.vanderkrol@wur.nl)

Supplementary Figures


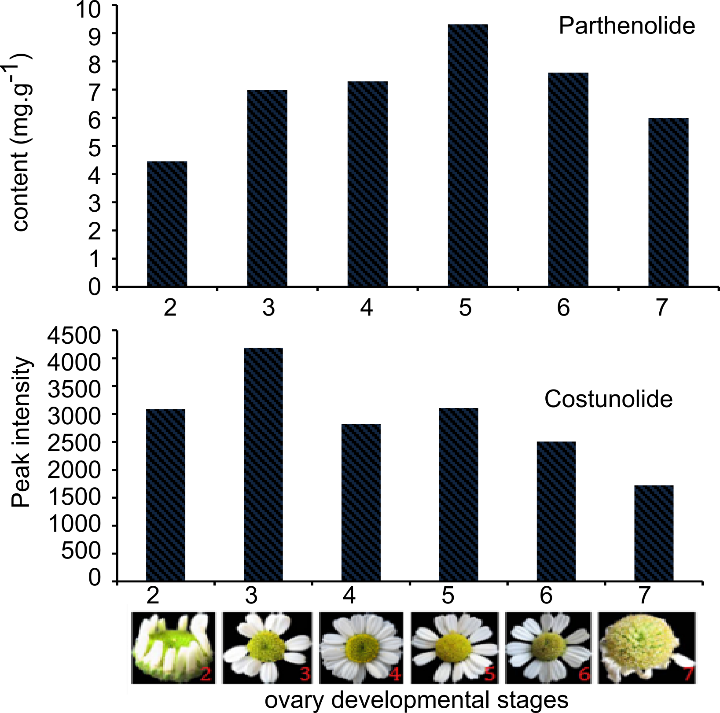


**Supplementary Figure 1- Accumulation of costunolide and parthenolide content of feverfew flowers in different developmental stages**

| 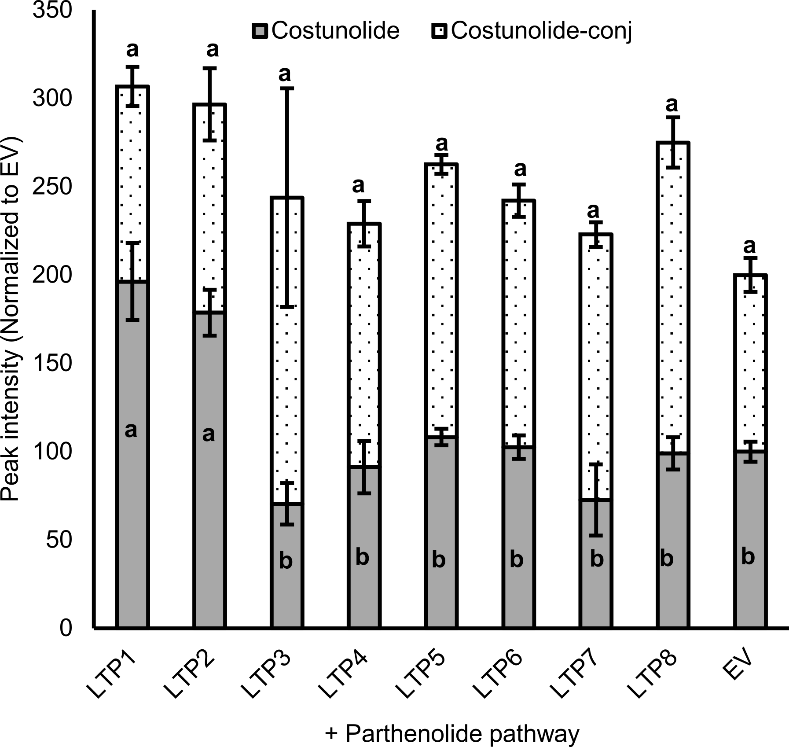 |
| --- |
| **Supplementary Figure 2. Effect of individual *Tp*LTPs on total costunolide and costunolide-conj accumulation upon transient co-expression with parthenolide pathway genes in *N. benthamiana*.** All values are normalized to EV (empty vector) and EV values for each compound is set at 100; costunolide (grey bars) and costunolide-conj (black-dotted bars). Means followed by a different letter are significantly different (P ≤ 0.05). |

| 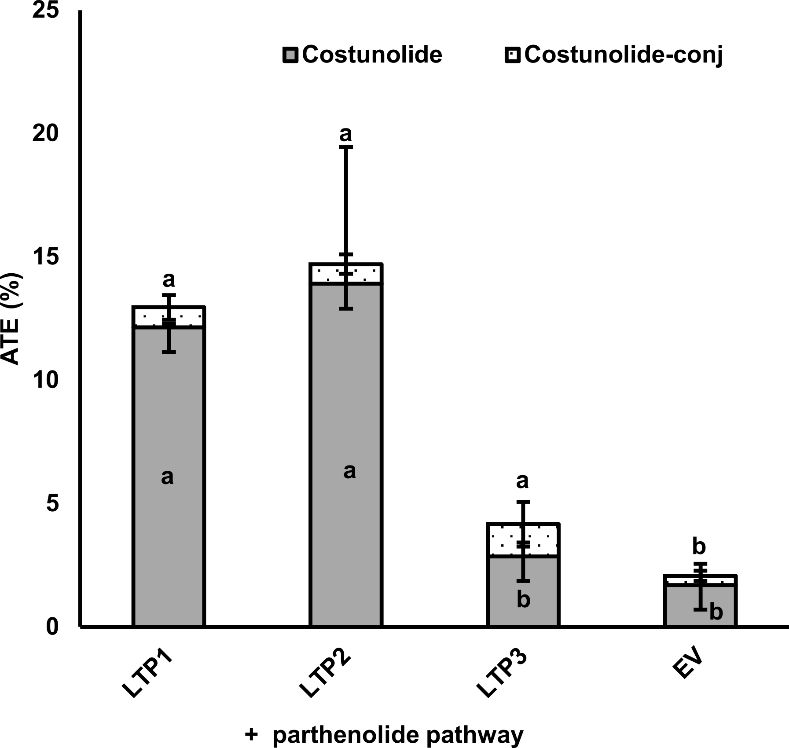 |
| --- |
| **Supplementary Figure 3. Costunolide apoplast transport efficiency (ATE) upon transient co-expression with the parthenolide pathway genes in *N. benthamiana*.** Only free costunolide accumulates in the apoplast upon co-expression of *Tp*LTP1 and *Tp*LTP2. Means followed by a different letter are significantly different (P ≤ 0.05). |

| 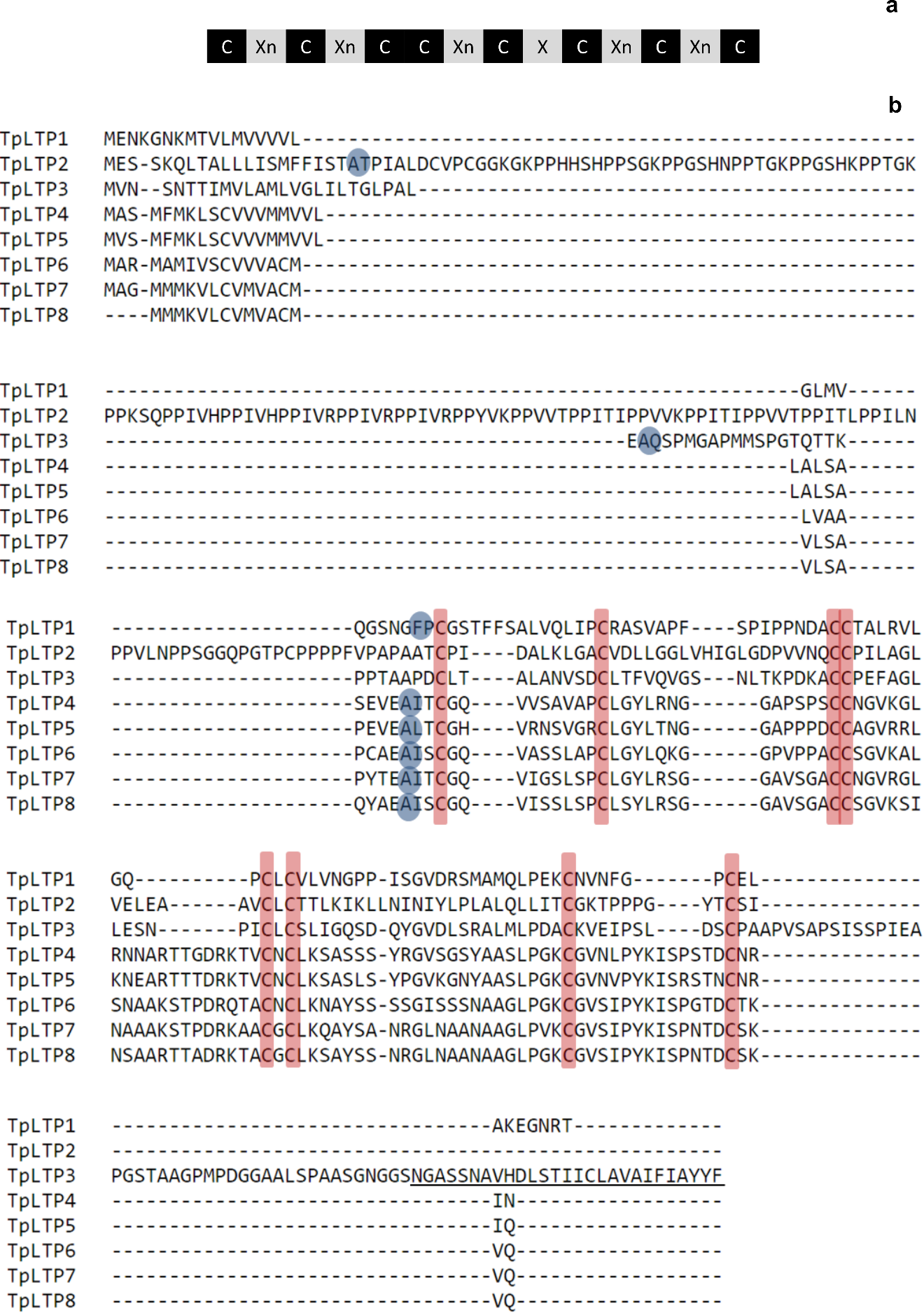 |
| --- |
| **Supplementary Fig. 4. Alignment of 8 *T. parthenium* lipid transfer proteins. (a) Characteristic structure of LTPs.** Conserved 8 cysteine residues (C). X represents amino acid residue and Xn represents a number of amino acid residues. **(b)** Multiple sequence alignment was done by Promals3d (<http://prodata.swmed.edu/promals3d/promals3d.php>). Blue indicates the secretion signal peptide, predicted by SignalP 4.1 (<http://www.cbs.dtu.dk/services/SignalP/>). 8 Cysteine motifs are indicated in red. The GPI anchor of *Tp*LTP3 is underlined, as predicted by PredGPI (<http://gpcr.biocomp.unibo.it/predgpi/pred.htm>). |

**Supplementary Tables**

| **Supplementary Table 1. Predicted log D values (corrected for pH) of studied compounds in this study.** artemisinic acid, dihydroartemisinic acid and some known phytohormones. Lower and negative values represent higher affinity for partitioning in the aqueous phase. Colours are designated by conditional formatting where the dark red represents greater positive values and the dark blue indicates the lower negative values. | | |
| --- | --- | --- |
| **Compound** | **logD ph5.5** | **logD pH 7.4** |
| Carlactone | 4.66 | 4.66 |
| Caryophyllene | 6.47 | 6.47 |
| Artemisinic acid | 3.58 | 1.79 |
| Costunolide | 3.36 | 3.36 |
| Dihydroartemisinic acid | 3.32 | 1.52 |
| Brassinolide | 3.18 | 3.18 |
| 5-Deoxystrigol | 2.64 | 2.64 |
| Parthenolide | 2.61 | 2.61 |
| 1-Naphthaleneacetic acid | 1.73 | -0.06 |
| Orobanchol | 1.41 | 1.41 |
| Abcisic Acid | 0.93 | -0.87 |
| Jasmonic Acid | 0.72 | -1.08 |
| 3-Indole acetic acid | 0.68 | -1.12 |
| Kinetin | -0.33 | 0.41 |
| Zeatin | -0.99 | -0.21 |
| Sucrose | -3.57 | -3.57 |

| **Supplementary Table 2. Primers used in this study.** | |
| --- | --- |
| **Name primer** | **5’ to 3’ sequence** |
| *Tp*LTP1-Forward | AAACTAGATCTGAATAATGGAAAACAA |
| *Tp*LTP1-Reverse | GACaCaACAATGCTTAACAATAATTC |
| *Tp*LTP2-Forward | GACACCAATACACATTGAGTGACA |
| *Tp*LTP2-Reverse | GGAAAACTTATATCCTTAAACACTCCA |
| *Tp*LTP3-Forward | CACCAAATATGGTTAATTCAAA |
| *Tp*LTP3-Reverse | TTAGAAGTAGTATGCTATGAAG |
| *Tp*LTP4-Forward | CACCAATGGCATCCATGTTC |
| *Tp*LTP4-Reverse | TCAATTAATCCTGTTGCAGTCAGT |
| *Tp*LTP5-Forward | TGTCCATGTTCATGAAGTTATC |
| *Tp*LTP5-Reverse | TTAGTGCTATAGTTTGCTTCCTTA |
| *Tp*LTP6-Forward | CAGCCCCCATGCATAAGTATCACA |
| *Tp*LTP6-Reverse | TCATGTGTAGTCGTGGCTTGCATGTTGG |
| *Tp*LTP7-Forward | CACACGCCAAGTGAATATCCATGC |
| *Tp*LTP7-Reverse | ATGTGTTATGGTGGCGTGCATGGTGTTG |
| *Tp*LTP8-Forward | ATTCGAACATTGGCAGGAATGA |
| *Tp*LTP8-Reverse | ACATTGAAGAGGCCAACCTCAC |
| 22251 3' RACE | ATCAGCCGATCTACCAACTGCAACAGGA |
| 22251 5' RACE | TTACGGTCCGTAGTGGTTCGTGCTTCAT |
| *Tp*LTP2 3' RACE | GATTACGCCAAGCTTTGGCAAGACTCCACCTCCTGGTTACACT |
| *Tp*LTP2 5' RACE | GATTACGCCAAGCTTGCATGTGGCTGCTGGAGCTGGCACAA |
| *Tp*LTP8 3' RACE | GGCGTGTTGCAGCGGCGTTAAGAGTATT |
